# The UPS in diabetes and obesity

**DOI:** 10.1186/1471-2091-9-S1-S6

**Published:** 2008-10-21

**Authors:** Simon S Wing

**Affiliations:** 1 Polypeptide Laboratory, Division of Endocrinology and Metabolism, Department of Medicine, McGill University and the McGill University Health Centre, Montreal, Quebec, H3A 2B2, Canada

## Abstract

Type 2 diabetes is caused by defects in both insulin signaling and insulin secretion. Though the role of the ubiquitin proteasome system (UPS) in the pathogenesis of type 2 diabetes remains largely unexplored, the few examples present in the literature are interesting and suggest targets for drug development. Studies indicate that insulin resistance can be induced by stimulating the degradation of important molecules in the insulin signaling pathway, in particular the insulin receptor substrate proteins IRS1, IRS2 and the kinase AKT1 (Akt). In addition, a defect in insulin secretion could occur due to UPS-mediated degradation of IRS2 in the β-cells of the pancreas. The UPS also appears to be involved in regulating lipid synthesis in adipocytes and lipid production by the liver and could influence the development of obesity. Other possible mechanisms for inducing defects in insulin signaling and secretion remain to be explored, including the role of ubiquitylation in insulin receptor internalization and trafficking.

Republished from Current BioData's Targeted Proteins database (TPdb; ).

## Protein pathway involvement in disease

### Introduction

The global incidence of diabetes mellitus (commonly referred to as diabetes) is increasing at such a rate as to be characterized as an epidemic. This is primarily due to changes in lifestyle that have led to obesity, a major risk factor for diabetes. In both developed and many developing societies, people have become more sedentary, thereby expending fewer calories. They have also adopted diets with greater calories, resulting in a net positive caloric balance stored in the body as fat (reviewed in [1,2]).

Diabetes is a metabolic disorder, the primary manifestation of which is elevated circulating blood glucose levels. Levels of blood glucose are normally maintained within a tight range of around 3.6–6.0 mM. Elevation of blood glucose, such as occurs after a meal, triggers the release of insulin from the β-cells of the islets of Langerhans in the pancreas. The circulating insulin has two major effects. One is to stimulate glucose uptake into cells by promoting the recruitment of a specific isoform of glucose transporter, GTR4 (GLUT4), from intracellular vesicles to the plasma membrane [3]. GTR4 is primarily expressed in skeletal muscle and adipose tissue [4]. Skeletal muscle accounts for the majority of insulin-stimulated glucose disposal after a meal, due to its larger overall mass. The other major effect of insulin is to suppress glucose production by the liver. The production of glucose by the liver is crucial for maintaining blood glucose levels in the fasted state and occurs by the breakdown of glycogen stores. Glucose can also be produced by the liver via *de novo *synthesis from amino acids and glycerol generated by the breakdown of protein stores in skeletal muscle and fat stores in adipose tissue, respectively (reviewed in [5]).

Although diabetes is best recognized as a disorder of glucose homeostasis, numerous other metabolic abnormalities are concomitant. Insulin is an anabolic hormone and thus stimulates protein synthesis and lipogenesis and inhibits protein degradation and lipolysis in target tissues. Therefore, diabetes is characterized not only by elevated blood glucose, but also by elevated levels of circulating amino acids and free fatty acids (reviewed in [6-8]).

### Classification of diabetes

Most cases of diabetes fall into one of two categories, which are very distinct in their etiologies and pathogenesis [9]. Type 1 diabetes, which accounts for around 10% of cases in most populations, is due to an autoimmune-mediated destruction of the insulin-producing β-cells in the pancreatic islets of Langerhans. When approximately 80–90% of β-cells are destroyed, diabetes becomes clinically evident and patients require insulin replacement therapy. Although typically occurring in youth (hence formerly called juvenile onset diabetes), type 1 diabetes can also occur in adults.

Type 2 diabetes accounts for the vast majority of the remaining cases of diabetes. This form of diabetes generally arises from the concurrent presence of two defects, insulin resistance, which is due to a defect in insulin signaling in target tissues, and a relative defect in pancreatic insulin secretion. The requirement for a defect in insulin secretion is demonstrated by obese insulin-resistant subjects. These patients do not develop diabetes because they are capable of secreting extremely high levels of insulin, probably by increasing their β-cell mass. Since obesity commonly results in insulin resistance, the worldwide epidemic of obesity has produced a concomitant epidemic of type 2 diabetes. Fat is now known to secrete humoral factors and some of these (e.g. leptin, adiponectin and resistin) can modulate insulin sensitivity; thus, the altered production of these adipokines in obesity could be involved in predisposition to diabetes (reviewed in [10]).

Other, rare forms of diabetes exist. For example, mutations in the insulin receptor can lead to severe insulin resistance and clinical defects such as leprechaunism [11]. Mutations in mitochondrial DNA have also been associated with diabetes [12]. Moreover, certain drugs can induce hyperglycemia or exacerbate pre-existing diabetes, as well as unmask latent forms of this disease.

### Mechanisms of insulin signaling

**Figure 1:**
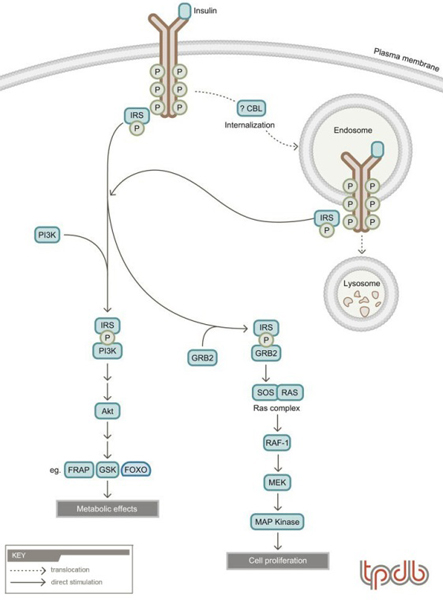
**General scheme of insulin signaling**. Insulin binding to its receptor activates the receptor tyrosine kinase activity. This leads to autophosphorylation of the receptor and recruitment and phosphorylation of insulin receptor substrate (IRS) proteins. Subsequently, other signaling molecules such as PI3-kinase (PI3K) and GRB2 are recruited, which leads to the activation of the Akt signaling pathway (which mediates many metabolic effects) and the MAP kinase pathway (which mediates cell proliferation), respectively. Insulin binding also leads to receptor internalization into endosomes. From endosomes, receptors can recycle back to the plasma membrane or traffic via multivesicular bodies to the lysosome for degradation. Phosphorylated IRS and the IRS-PI3K complex are signaling molecules that can be inactivated by degradation through the ubiquitin proteasome system. Receptor internalization and trafficking between the endosome and lysosome has been shown to be dependent on ubiquitylation for other receptor tyrosine kinases, but the requirement for UBIQ (ubiquitin) in the case of the insulin receptor remains speculative. In some adipocyte cell lines, the ubiquitin protein ligase CBLB (Cbl-b) has been implicated in mediating translocation of the GTR4 (GLUT4) glucose transporter to the plasma membrane via a RHOQ (Tc10)-dependent pathway.

Circulating insulin exerts its effects by binding to insulin receptors on target tissues, leading to the activation of multiple signaling pathways (Figure 1) (reviewed in [13]). The insulin receptor is a membrane receptor tyrosine kinase and binding of insulin to this receptor induces a conformational change, which activates the tyrosine kinase activity on the cytoplasmic surface of the cell [14]. This results in both autophosphorylation of the receptor and phosphorylation of other protein molecules. The immediate substrates of the insulin receptor kinase are members of the insulin receptor substrate (IRS) family (reviewed in [15]). These IRS family members vary in their relative importance in different cells and tissues and therefore can mediate specific actions of insulin. Phosphorylation of IRS proteins on tyrosine residues results in the recruitment of signaling molecules to these proteins. This recruitment is typically mediated by phosphotyrosine binding motifs such as SH2 (Src homology 2) domains and a key such signaling molecule is PI3-kinase. Recruitment of PI3-kinase to IRS1 (IRS-1) results in the phosphorylation of membrane phosphoinositides [16], which leads to membrane recruitment and activation of a downstream signaling kinase, AKT1 (Akt). Akt kinase, in turn, phosphorylates other proteins, the identities of which are largely unknown. However, several downstream targets of AKT1 activation have been identified including FRAP (mTOR) [17] and GSK [18], which lead to stimulation of protein [19] and glycogen synthesis, respectively. Ultimately, these changes result in the metabolic effects of insulin as described above.

In cell culture, and importantly during fetal development, insulin is a growth factor. The stimulation of cell proliferation by insulin is mediated through the activation of the Ras-Raf-MAP kinase pathway [20-23]. Activation of this pathway occurs due to the ability of insulin-stimulated IRS1 to bind the GRB2 (Grb2) protein in proliferating cells. This, in turn, recruits and activates the guanine nucleotide exchange factor SOS (Sos), leading to the activation of the Ras-Raf-MAP kinase signaling pathway [20-23].

### Mechanisms of insulin secretion

**Figure 2:**
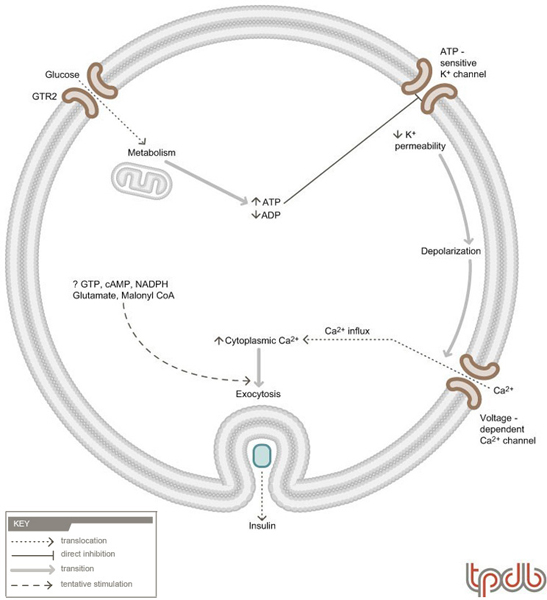
**General scheme of insulin secretion**. In the β-cells of the pancreas, elevated blood glucose leads to increased levels of intracellular glucose-6-phosphate.  Metabolism of the glucose-6-phosphate through glycolysis and mitochondrial metabolism, leads to increased levels of ATP and to lower levels of ADP. This sequentially results in inhibition of an ATP-sensitive K^+ ^channel, depolarization of the plasma membrane, activation of a voltage-dependent Ca^2+ ^channel, influx of Ca^2+ ^and exocytosis of insulin. Mitochondrial metabolism participates in this process, not only by generating ATP, but also by modulating levels of other metabolites (e.g. GTP, cAMP, NADPH, glutamate, malonyl CoA), which might also contribute to glucose-stimulated insulin secretion. The ATP-sensitive K^+ ^channel and voltage-dependent Ca^2+ ^channel are sites that could be modulated by the ubiquitin proteasome system (UPS). In addition, β-cell survival can be impaired by UPS-mediated degradation of IRS2 in these cells (see text for details).

Insulin secretion is tightly controlled by levels of circulating glucose and to a lesser extent, amino acids (Figure 2) (reviewed in [24,25]). The ability of pancreatic β-cells to act as a glucose sensor is mainly due to the expression of specific isoforms of the glucose transporter and glucokinase in these cells. β-cells express the GTR2 (GLUT2) isoform of glucose transporter, which has a Km for glucose transport of 15–20 mM. Thus, glucose flux into these cells is dependent on circulating concentrations of glucose. In addition, the β-cell glucokinase has a Km of around 8 mM, which also allows generation of glucose-6-phosphate to be dependent on ambient glucose levels. Therefore, high levels of glucose generate more glucose-6-phosphate, which, through glycolysis and oxidative phosphorylation, leads to the production of ATP from ADP. This increased intracellular ratio of ATP to ADP results in the inhibition of an ATP-sensitive K^+ ^channel in the plasma membrane. Decreased efflux of K^+ ^leads to depolarization of the plasma membrane, which activates a voltage-dependent Ca^2+ ^channel. Ca^2+ ^results in exocytosis of insulin-containing secretory granules and the release of insulin into the circulation. The mitochondrial metabolism also generates several other metabolites that appear to be able to stimulate insulin secretion beyond the effects of the adenine nucleotides. Chronic hyperglycemia also activates transcription of the insulin gene, resulting in *de novo *synthesis of insulin.

### Ubiquitylation and insulin resistance

The molecular mechanisms that result in cells becoming resistant to insulin are only beginning to be defined [26]. In cell culture, resistance can occur due to downregulation of insulin receptors. In patients with diabetes, a small number of studies indicate that receptor numbers are decreased in isolated strips of abdominal skeletal muscle [27], but normal in hepatocytes isolated from liver biopsies [28]. More consistent are observations of decreased receptor kinase activity in these samples, measured as the degree of insulin-stimulated tyrosine phosphorylation of the receptor and IRS1 [27,28]. Most of the insulin resistance appears to be due to resistance at signaling molecules downstream of the receptor. For example, in insulin resistant states, IRS1 can become phosphorylated on serine residues [29], reviewed in [30]. This serine phosphorylation is associated with decreased insulin-stimulated tyrosine phosphorylation of IRS1 resulting in the decreased ability of this protein to mediate signaling. Interestingly, IRS activity can also be lowered by degradation. In various cell lines (for example CHO [31], MEF and NIH3T3 [32]), both IRS1 and IRS2 (IRS-2) can be ubiquitylated and degraded by the proteasome. This ubiquitylation appears to be mediated by the proteins SOCS1 and SOCS3 [33], which function as substrate recognition factors in the cullin-RING ligases subset of ubiquitin protein ligases. Overexpression of these proteins stimulates degradation of IRS1 and IRS2 in cultured cells and in mouse liver [33]. The SOCS1 and SOCS3 proteins interact with the von Hippel Landau (VHL) subfamily of cullin-RING ligases and are induced by cytokines [34]. Such induction by inflammatory factors is particularly interesting in light of numerous recent observations suggesting that diabetes and obesity are pro-inflammatory states and that inflammation plays an important role in mediating insulin resistance (reviewed in [35]).

Other studies also support a role for modulation of IRS levels in mediating insulin resistance. For example, nitric oxide (NO) has been implicated in mediating insulin resistance, as iNOS knockout mice are more sensitive to insulin and more resistant to diet-induced obesity than wild-type mice [36]. A possible mechanism for these effects is demonstrated by the induction of IRS1 degradation by iNOS and NO in cultured muscle cells [37]. In addition, other cell culture models of insulin resistance, such as osmotic stress or chronic exposure to insulin or IGF1 (IGF-1), have been shown to promote degradation of IRS2 in NIH3T3 cells [32]. IRS1 degradation also occurs upon chronic insulin exposure of CHO cells that overexpress the insulin receptor or IRS1 [31]. The exact mechanisms and proteolytic system(s) responsible for the degradation of IRS proteins in these latter situations remain unknown. Nonetheless, it is clear that degradation of IRS proteins can be a contributing factor in the development of insulin resistance.

In addition, recent data suggest that resistance could also occur more distally at the level of AKT1 due to loss of activity of this signaling molecule. For example, stimulation of NIH3T3 adipocytes by the cytokine TNFA (TNFα) leads to the ubiquitylation and loss of AKT1. Interestingly, this ubiquitylation of AKT1 appears to be dependent on the activation of caspase 6, as ubiquitylation is blocked by an inhibitor of this caspase [38]. Finally, as distal parts of the signaling pathway become better defined, new roles for ubiquitylation become identified. Insulin usually suppresses hepatic glucose production, and a key feature of the insulin resistance in type 2 diabetes is increased glucose output from the liver via gluconeogenesis. Insulin exerts this effect by suppressing transcriptional activation of genes encoding enzymes in the gluconeogenic pathway. Recent findings indicate that insulin stimulates the phosphorylation of one of these transcriptional activators, TORC2, and this phosphorylation leads to its ubiquitylation and targeting for degradation by the COP1 ubiquitin protein ligase [39].

Another possible mechanism for modulating insulin signaling is through the regulation of insulin receptor trafficking. Like other membrane receptors, ligand binding results in internalization of the receptor, which can either be recycled to the plasma membrane or targeted to the lysosome for degradation. During this trafficking through the cell in endosomes, the receptor remains active [40] and so increased or more rapid trafficking could result in attenuated signaling. In cell lines, such as HeLa and HEK293, many receptor tyrosine kinases are known to be ubiquitylated upon ligand stimulation and such ubiquitylation is clearly involved in downregulation of the receptor by stimulating the trafficking of the receptor from the plasma membrane to the lysosome (reviewed in [41]). Indeed, interfering with the downregulation of the receptor (as occurs in cells with mutant forms of the receptor) or the ubiquitin protein ligase (thus preventing their interaction), results in increased levels of the receptor. In the case of EGF [42,43] or HGF [44], this can lead to oncogenic transformation of the cells. Although multiple receptors such as the EGF, PDGF and HGF receptors are known to be ubiquitylated, whether ubiquitylation of insulin or IGF1 receptors occurs under similar cell culture conditions remains controversial, with conflicting observations for these very similar receptors [45,46]. However, this does not preclude a role for ubiquitylation in downregulation of the insulin receptor, since other non-receptor proteins are also ubiquitylated as part of the trafficking process. For example, CBLB (Cbl-b) is a ubiquitin protein ligase that ubiquitylates several receptors upon ligand stimulation and loss of this protein's ability to do so increases signaling [42,44,47] (reviewed in [48]). Interestingly, characterization of mice deficient in the CBLB isoform reveals that these mice are protected from diet-induced obesity and insulin resistance [49]. Thus, this loss of CBLB activity could be stabilizing the insulin receptor and/or increasing its signaling; however, this is yet to be clearly demonstrated.

### Ubiquitylation and insulin secretion

In contrast to the role of ubiquitylation in insulin action, the role of the UPS in insulin secretion is less well defined. Inhibitors of the proteasome can acutely enhance glucose-stimulated insulin release from rat islets in culture [50], but appear to have the opposite effect in a mouse β-cell line [51]. Accumulation of ubiquitylated proteins, with evidence of associated inhibition of proteasome activity, has been described in cultured islets and β-cells [52] and observed in the pancreas of the Zucker diabetic rats [53], but whether this is a cause or a consequence of β-cell damage remains unclear. Few specific substrates have been identified. Levels of the ATP-sensitive K^+ ^channel [54] and the voltage-dependent Ca^2+ ^channel [51] could be regulated by the UPS. One intriguing substrate is IRS2, which is required for β-cell survival and is degraded in the INS1 (INS-1) β-cell line upon chronic exposure to hyperglycemia or IGF1. The hyperglycemia stimulates the phosphorylation of IRS2 on serine and threonine and this phosphorylation appears to target the protein for degradation by the proteasome, as proteasome inhibitors block this degradation [55]. Whether SOCS1 or SOCS3 are involved in the ubiquitylation of IRS2 in these cells remains unknown. Thus, chronic hyperglycemia could employ this mechanism to decrease IRS2 and induce β-cell apoptosis. Recently, the TNAP3 (A20) protein has been shown to be induced in islets undergoing apoptosis and to protect these cells from death [56]. TNAP3 is a dual function protein containing both deubiquitylating and ubiquitin ligase activities, although the exact mechanism of the action of this protein in this situation remains unclear.

### Ubiquitylation and biological actions of insulin

In previous sections within *Protein pathway involvement in disease*, the roles of ubiquitylation in modulating factors that could be involved in causing diabetes were discussed. Ubiquitylation also plays various roles in mediating the actions of insulin. As described earlier, insulin has functions other than stimulation of glucose uptake and suppression of gluconeogenesis. Insulin stimulates lipogenesis in fat, as well as the liver, by activating acetyl-coA carboxylase, the rate-limiting enzyme in fatty acid synthesis [57]. Upon fasting, lipogenesis is inhibited and acetyl-coA carboxylase is inactivated as a result of low insulin levels, as well as the effects of catecholamines and glucagon. However, acetyl-coA carboxylase has also been shown to be inactivated by degradation in adipose tissue. Upon fasting, levels of the pseudokinase protein TRIB3 (Tribbles3) increase in murine fat and the protein associates with the ubiquitin protein ligase COP1, thus stimulating the ubiquitylation and degradation of acetyl-coA carboxylase [58]. Mice overexpressing TRIB3 in adipose tissue are protected from diet-induced obesity.

Fat is normally transported in the blood in esterified form as triglycerides and packaged with protein into lipoprotein particles. Synthesis of these apoprotein constituents of the particle appears to be important in determining the levels of lipoprotein particles and fat circulating in the blood. Apoprotein B48 (apoB48) is the major apoprotein in the VLDL and LDL particles, which are the major circulating forms of lipid in the blood. Like most secreted proteins, apoB48 enters the lumen of the ER following synthesis. However, in human liver carcinoma HepG2 cells, a significant fraction of synthesized apoB48 is exported from the ER lumen to be degraded by the ER-associated degradation pathway [59]. This pathway involves ubiquitylation of the exported proteins and degradation by the proteasome. Interestingly, exposure of the cells to proteasome inhibitors can inhibit degradation of apoB48, resulting in increased secretion of the lipoprotein containing it. Dietary omega-3 fatty acids are known to lower levels of VLDL and, in intestinal explants from gerbils, this appears to be due to the ability of these fatty acids to stimulate degradation of apoB48 [60].

Insulin is well recognized to be anabolic in skeletal muscle, by stimulating protein synthesis and inhibiting protein degradation. Protein degradation in skeletal muscle is dependent on the UPS. The cellular regulatory mechanisms have actually been best described for IGF1, but these mechanisms are also likely to apply to insulin, as the signaling pathways of these proteins are very similar. IGF1 activates AKT1, which leads to the activation of FRAP (mTor), the translation initiation factor IF4B (eIF4B) and KS6B1 (p70S6) kinase, resulting in the stimulation of protein synthesis [17]. Simultaneously, AKT1 activation leads to phosphorylation of members of the FOXO transcription factors, which results in their exclusion from the nucleus [61]. Nuclearly localized FOXO transcription factors normally activate the transcription of two important ubiquitin protein ligases, TRI63 (MuRF1) and FBX32 (atrogin-1, MaFBx). In skeletal muscle, the increased expression of these ligases is tightly associated with protein catabolism and inactivation of the genes coding for these proteins in the mouse leads to blunted muscle atrophy [62]. Thus, growth factor-stimulated cytosolic retention of FOXO factors turns off protein degradation.

Increased expression of components of the ubiquitin proteasome pathway has been observed in the muscles of patients in catabolic states [63-66] but not in all situations. This is probably due to the activation of protein expression when the wasting process is initiated and might not be present in very early [67] or late stages of disease. Some of the suppressive effects of insulin might be related to possible interactions between IDE, an enzyme that could degrade insulin, and the proteasome [68].

### Ubiquitin and ubiquitin-like proteins and type 1 diabetes

As described earlier within the section *Protein pathway involvement in disease*, type 1 diabetes arises from an immunological destruction of the insulin producing β-cells. Although an environmental trigger is suspected in this disease, a genetic predisposition to type 1 diabetes is well established, with the HLA haplotype the major determinant of risk, although other genes are also involved. It has also been shown that the M55V substitution in the *SUMO4 *gene is associated with the risk of developing type 1 diabetes [69] (reviewed in [70]). SUMO4 can be conjugated to the IKBA (IκBα) inhibitor of NFκB, leading to the inhibition of NFκB activation. Expression of the M55V variant of SUMO4 in human liver carcinoma HepG2 cells results in a 5.5-fold increase in NFκB transcriptional activity compared with expression of the wild-type form of this protein [69]. This increased activity can lead to increased cytokine secretion, which could play a role in the immune destruction of the β-cells.

## Disease models, knockouts and assays

Commonly used animal models of type 2 diabetes include the ob/ob and db/db mice, which are deficient in leptin or the leptin receptor, respectively [71]. Common rat models include the lean (wild-type) and obese (heterozygous) fatty Zucker rats, which possess a leptin receptor mutation and are normoglycemic [72]. Homozygous mutant rats are obese and become diabetic. Although common forms of human obesity are not due to such defects in leptin or its receptor, obese subjects have generally been found to be leptin-resistant. High fat feeding will readily induce obesity and diabetes in C57bl/6 mice and these mice are frequently used as animal models of overfeeding [73]. The laboratories of C.R. Kahn and M. White at Harvard Medical School have been prominent in generating tissue-specific knockouts of the insulin receptor and insulin receptor substrates, respectively. CBLB (Cbl-b)-deficient mice were created by the D. Bowtell laboratory at the Peter MacCallum Cancer Institute. A larger listing of animal models of diabetes has been recently published [74].

## Disease targets and ligands

As of February 2008, evidence for the involvement of the UPS in the pathogenesis of diabetes remained limited. The downregulation of key signaling molecules such as IRS1, IRS2 and AKT1 (Akt) in insulin resistant states and the potential roles of the SOCS1 and SOCS3 proteins in targeting these signaling molecules to ubiquitin-dependent degradation is clearly intriguing. Similarly, the high glucose stimulated degradation of IRS2, which plays an important role in the survival of the insulin secreting β-cells, is also interesting. Pharmacological inhibition of the ligases responsible for this degradation would be of interest. The increased insulin sensitivity in CBLB (Cbl-b) knockout mice suggests that this ligase could also be a potential target in spite of the lack of evidence that CBLB ubiquitylates and downregulates the insulin receptor.

## New frontiers in drug discovery

As of February 2008 there were only a limited number of studies implicating the UPS in the pathogenesis of diabetes and obesity. The most striking observations of these investigations are that the UPS has a role in downregulating IRS proteins and thereby contributing to the two main defects in diabetes, insulin resistance and impaired insulin secretion. Reversing these effects could be a novel approach in the treatment of diabetes and might be tested using gene inactivation models of the SOCS1 and SOCS3 proteins. Such establishment of a clear role for these proteins could identify them as targets for drug therapy in the treatment of diabetes. In view of the pervasiveness of the involvement of the UPS in cell signaling and receptor internalization and trafficking, it is likely that many more roles for the UPS in these disorders will be uncovered, leading to novel drug targets. For example, drug inhibition of CBLB (Cbl-b) or of proteins that are involved in downregulation of the insulin receptor via the endosomal lysosomal system would be an interesting approach to explore. The recent developments of large scale screening technologies will hasten the pace of such discoveries. For example, shRNA libraries against UPS genes have been constructed [75] and could be used to screen for alterations in insulin action in cultured cells.

## Note added in proof

Recently, Xu *et al.* described a CUL7 ubiquitin ligase complex that also binds and targets IRS1 for ubiquitylation and degredation [76].

## List of abbreviations used

EGF: epidermal growth factor; HGF: hepatocyte growth factor; iNOS: nitric oxide synthase; IRS: insulin receptor substrate; LDL: low density lipoprotein; PDGF: platelet derived growth factor; SOCS: suppressor of cytokine signaling; VLDL: very low density lipoprotein.

## Competing interests

The author declares that he has no competing interests.

## Publication history

Republished from Current BioData's Targeted Proteins database (TPdb; ).
